# Reciprocal regulation between sirtuin-1 and angiotensin-II in the substantia nigra: implications for aging and neurodegeneration

**DOI:** 10.18632/oncotarget.5596

**Published:** 2015-09-10

**Authors:** Carmen Diaz-Ruiz, Ana I. Rodriguez-Perez, Daniel Beiroa, Jannette Rodriguez-Pallares, Jose L. Labandeira-Garcia

**Affiliations:** ^1^ Laboratory of Neuroanatomy and Experimental Neurology, Dept. of Morphological Sciences, CIMUS, University of Santiago de Compostela, Santiago de Compostela, Spain; ^2^ Networking Research Center on Neurodegenerative Diseases (CIBERNED), Madrid, Spain; ^3^ Department of Physiology, CIMUS, University of Santiago de Compostela, Santiago de Compostela, Spain; ^4^ CIBER Fisiopatología de la Obesidad y Nutrición (CIBERobn), Spain

**Keywords:** dopamine, longevity, neuroinflammation, parkinson, resveratrol, Gerotarget

## Abstract

Local angiotensin II (AII) and sirtuin 1 (SIRT1) play a major role in the modulation of neuroinflammation, oxidative stress and aging-related dopaminergic vulnerability to damage. However, it is not known whether the modulation is related to reciprocal regulation between SIRT1 and AII. In the present study, a single intraventricular injection of AII increased nigral SIRT1 levels in young adult rats. Although AII activity is known to be increased in aged rats, levels of SIRT1 were significantly lower than in young controls. Treatment with the SIRT1-activating compound resveratrol increased nigral SIRT1 levels in aged rats. Levels of SIRT1 were significantly higher in aged wild type mice than in AII type-1 receptor (AT1) deficient mice. In cell culture studies, treatment with AII also induced a transitory increase in levels of SIRT1 in the MES 23.5 dopaminergic neuron and the N9 microglial cell lines. In aged rats, treatment with resveratrol induced a significant decrease in the expression of AT1 receptors and markers of NADPH-oxidase activation (p47^phox^). In aged transgenic mice over-expressing SIRT1, levels of AT1 and p47^phox^ were lower than in aged wild type controls. In vitro, the inhibitory effects of resveratrol on AII/AT1/NADPH-oxidase activity were confirmed in primary mesencephalic cultures, the N9 microglial cell line, and the dopaminergic neuron cell line MES 23.5, and they were blocked by the SIRT1 specific inhibitor EX527. The present findings show that SIRT1 and the axis AII/AT1/NADPH-oxidase regulate each other. This is impaired in aged animals and may be mitigated with sirtuin-activating compounds.

## INTRODUCTION

The sirtuins are a highly conserved family of enzymes that regulate lifespan in lower organisms. The sirtuins act as NAD^+^-dependent protein deacetylases on a variety of targets, including histones, transcription factors and apoptotic modulators [1-4]. In mammals, the sirtuin family has seven members of (SIRT1-SIRT7). SIRT1 is present in the nucleus and cytoplasm and deacetylates several proteins involved in cell survival, metabolism and stress response, and it has been found to stimulate resistance to oxidative stress in several types of cells [5, 6]. SIRT1 and pharmacological SIRT1 activators such as resveratrol (RV) have been suggested to counteract the effects of aging [7-9]. Advancing age itself is the most significant risk factor for the development of neurodegenerative diseases such as Parkinson's disease (PD) [10, 11]. However, the effects of SIRT1 on brain senescence and the development of neurodegenerative diseases are not clear [12-14]. SIRT1-activating compounds such as resveratrol and quercetin exert neuroprotective effects on dopaminergic neurons in PD models [15, 16]. Initially, it was not clear whether the antioxidant or sirtuin-activating effect (or both) was responsible for the effects of resveratrol [15]. However, more recent studies have shown that the effect of SIRT1 on the microglial neuroinflammatory response plays a major role in the protective effects of resveratrol [16-18].

Angiotensin II (AII) is the most important effector peptide of the renin-angiotensin system (RAS), and its effects are mediated by two main cell receptors: AII type 1 and 2 (AT1 and AT2) receptors. AT2 receptors exert actions that are directly opposed to those mediated by AT1 receptors [19-20]. Hyperactivation of local or tissue RAS, via AT1 receptors and NADPH oxidase activation, mediates oxidative stress and several key events in inflammatory processes that play a major role in various aging-related diseases [21, 22]. Furthermore, local RAS has been associated with decreased longevity and age-related degenerative changes in a number of tissues [23-25]. In recent studies, we have demonstrated the presence of local RAS in the substantia nigra and striatum of rodents and primates [26-28], including humans [29]. We have also demonstrated that overactivation of local RAS, via AT1 receptors, exacerbates neuroinflammation, oxidative stress and dopaminergic cell death, all of which were inhibited by treatment with AT1 receptor antagonists [26, 30-32]. We have observed RAS overactivation in the nigrostriatal system of aged rats, along with enhanced levels of neuroinflammation and oxidative stress markers, leading to increased dopaminergic cell vulnerability to neurotoxins [33, 34], and possibly PD in humans [35-37].

The local RAS and SIRT1 both appear to play major roles in the modulation of neuroinflammation, oxidative stress and aging-related dopaminergic vulnerability to damage. However, it is not known whether SIRT1 and AII regulate each other in the modulatory process; manipulation of this reciprocal regulation may be useful for the development of neuroprotective treatments. In the present study, we used *in vivo* models (young and aged rats, and young and aged knockout and transgenic mice) and *in vitro* models (primary mesencephalic cultures and dopaminergic and microglial cell lines) to investigate a possible reciprocal regulation between SIRT1 and AII in the dopaminergic system.

## RESULTS

### Location of SIRT1 in dopaminergic neurons and glial cells in the rat substantia nigra, primary cultures and cell lines

**Figure 1:**
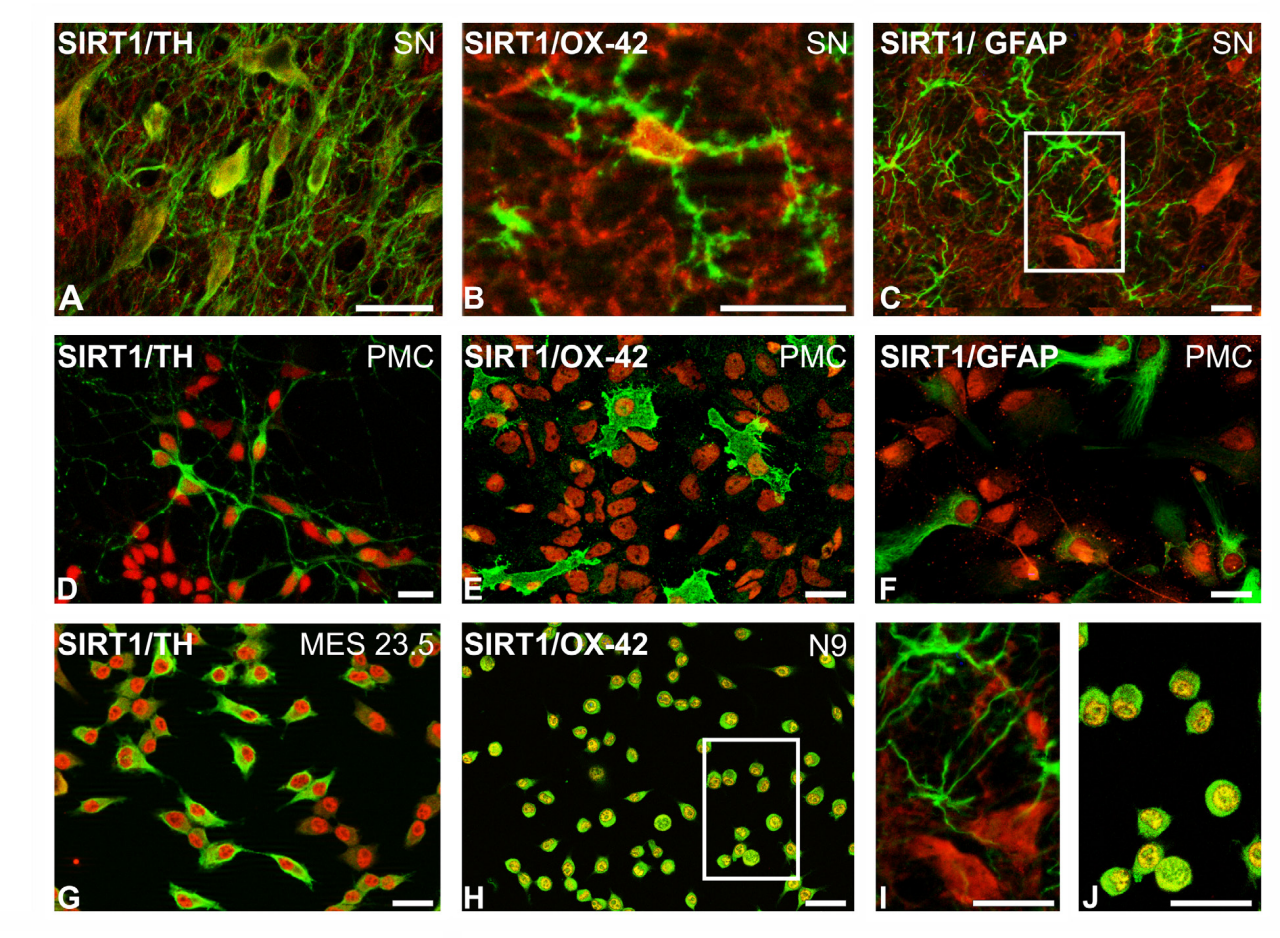
Double immunofluorescence and laser confocal microscopy showing co-localization (yellow) of the dopaminergic marker TH (green; A,D,G), the microglial marker OX-42 (green; B,E,H,J), or the astroglial marker GFAP (green; C,F,I) and SIRT1 (red) SIRT1 expression was observed in the nucleus and cytoplasm of dopaminergic neurons **A.**, **D.**, **G.** and microglial cells **B.**, **E.**, **H.**, **J.** in the substantia nigra **A.**, **B.**, primary cultures **D.**, **E.** and dopaminergic **G.** and microglial **H.**, **J.** cell lines. SIRT1 expression was observed in astrocytes in cultures **F.** but not clearly distinguished in astrocytes in the nigra of control rats **C.**, **I.** Boxed areas in C and H are magnified in I and J. Scale bars: 25μm. GFAP, glial fibrillary acid protein; TH, tyrosine hydroxylase; PMC, primary neuron-glia mesencephalic cultures.

Immunohistochemical analysis clearly showed SIRT1 expression in the nucleus and cytoplasm of dopaminergic neurons and microglial cells in the substantia nigra of control rats (Figure 1A, 1B). However, SIRT1 immunolabelling was not clearly distinguished in astrocytes in the substantia nigra of normal rats (Figure 1C, 1I). SIRT1 immunolabelling was observed in the nucleus and cytoplasm of dopaminergic neurons, microglia and astrocytes in primary cultures (Figure 1D-1F), suggesting that SIRT1 is upregulated in astrocytes under the *in vitro* conditions used. SIRT1 immunolabelling was also observed in the neuronal cell line MES 23.5 (Figure 1G) and the N9 microglial cell line (Figure 1H, 1J).

### Effect of RAS overactivity on SIRT1 expression

**Figure 2:**
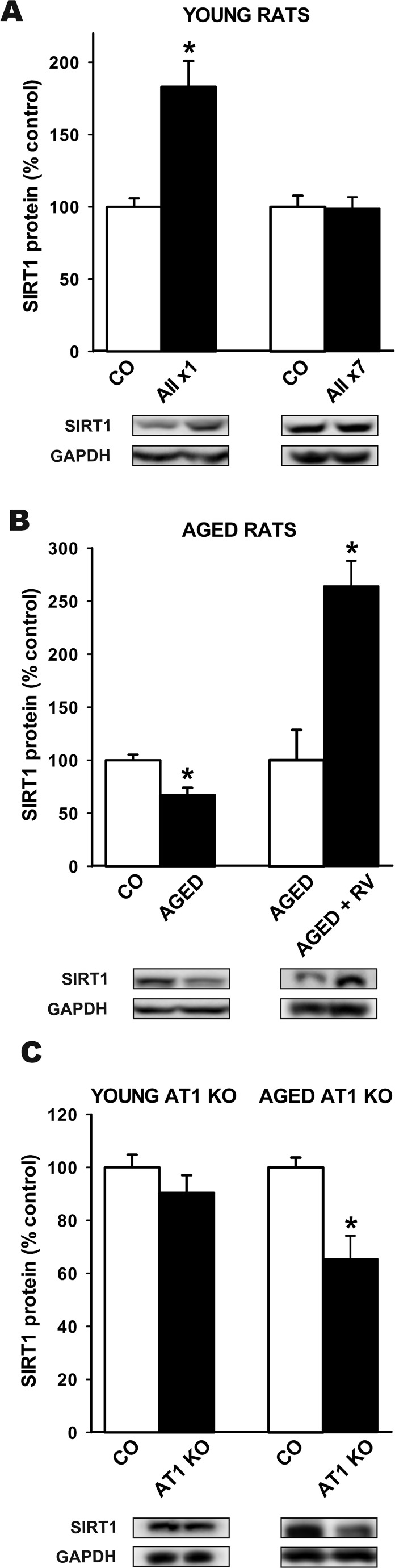
Western blot analysis of changes in the expression of SIRT1 in the nigral region In young adult rats after a single or seven intraventricular injections of AII or vehicle **A.**, aged rats relative to control (i.e. young) rats or aged rats treated with resveratrol **B.**, and in aged and young AT1 KO mice relative to wild type controls of the same age **C.** Protein expression was measured relative to the GAPDH band value. The results were normalized to the values for controls (100%). Data are means ± SEM. *p < 0.05 relative to controls (Student's *t* test). AII, angiotensin 2; x1, single injection; x7, 7 consecutive injections for 7 days; CO, control; RV, resveratrol.

In the nigral region of young adult rats that received a single intraventricular injection of AII, increased levels of SIRT1 protein were observed 16 h after the injection. However, no significant increase in SIRT1 protein levels was observed in young adult rats that received daily intraventricular injection of AII for 7 days (Figure 2A).

Activation of the AII/AT1 pathway is known to be enhanced in the substantia nigra of aged rats; see [33] for details. However, levels of SIRT1 in aged rats were significantly lower than in young controls. Interestingly, treatment with resveratrol significantly increased the levels of SIRT1 in the nigral region of aged rats (Figure 2B). We also compared SIRT1 levels in the nigral region of aged AT1 KO mice (18 months old) and wild type mice of the same age. Levels of SIRT1 were significantly higher in aged wild type mice than in AT1 KO mice (Figure 2C). However, no significant differences were observed between young wild type controls and young AT1 KO mice (Figure 2C).

**Figure 3:**
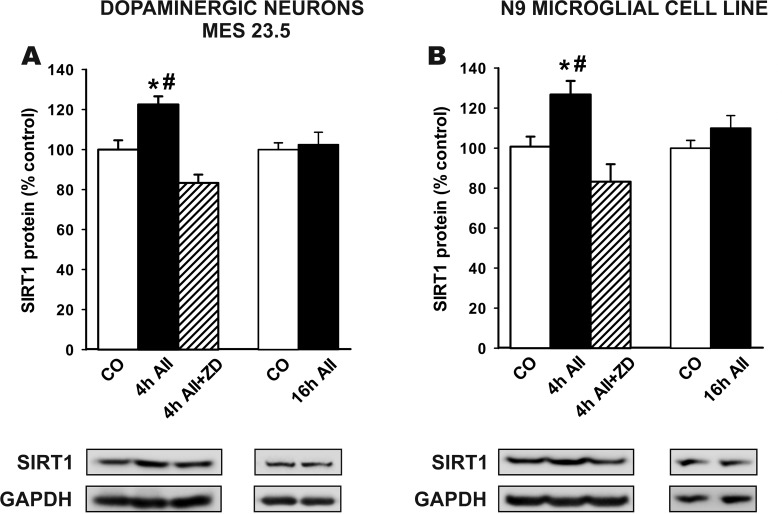
**Western blot analysis of changes induced by treatment with AII (100 nM) in the expression of SIRT1** in the dopaminergic cell line MES 23.5 **A.** and the N9 microglial cell line **B.** The AII-induced increase in SIRT1 expression was inhibited by the AT1 receptor antagonist ZD-7155. Protein expression was measured relative to the GAPDH band value. The results were normalized to the values for controls (100%). Data are means ± SEM. *p < 0.05 relative to controls; ^#^*p* < 0.05 relative to AII+ZD group (one-way ANOVA followed by Student-Newman Keuls). AII, angiotensin II; CO, control; ZD, AT1 antagonist ZD-7155.

Treatment of the MES 23.5 dopaminergic neuron cell line and the N9 microglial cell line with AII induced an increase in levels of SIRT1 4 h after treatment, but no significant increase was observed 16 h after treatment with AII. AII-induced increase in SIRT1 levels in dopaminergic cells and microglial cells was inhibited by treatment with the AT1 antagonist ZD 7155 (Figure 3).

### Effect of SIRT1 activation on RAS activity

**Figure 4:**
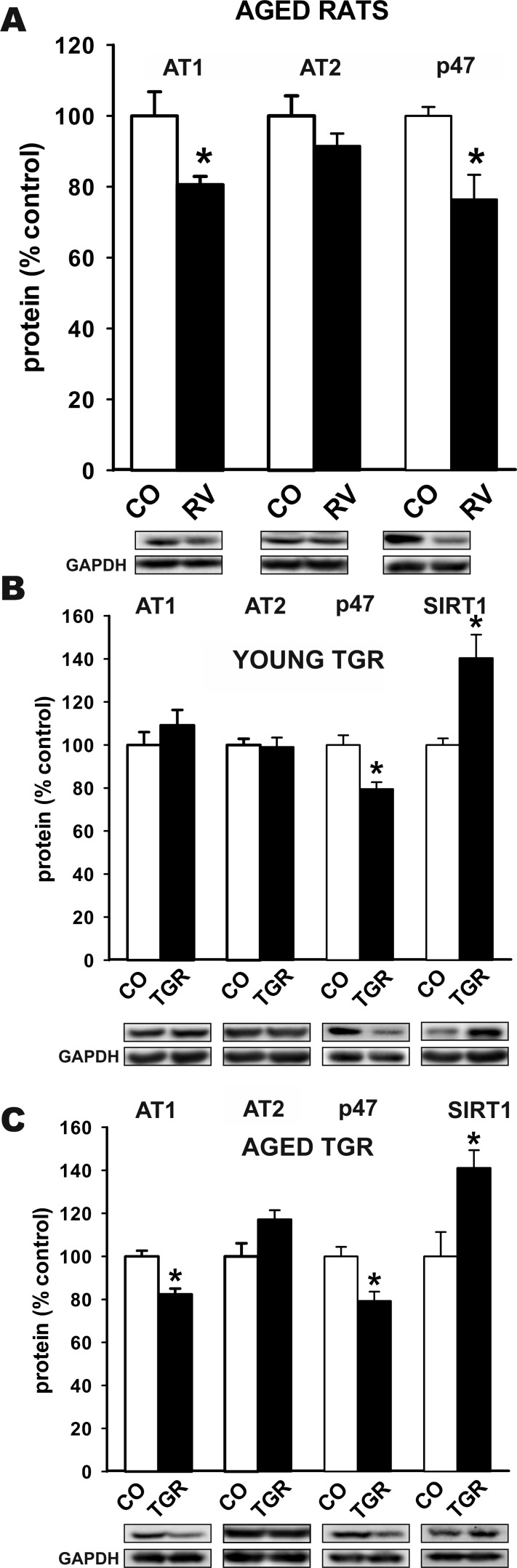
**Western blot analysis of changes in the expression of angiotensin receptors (AT1 and AT2) and NADPH-oxidase activation** (i.e. p47^phox^ subunit expression) in the nigral region in aged rats treated with resveratrol or vehicle **A.**, and in young **B.** and aged **C.** transgenic mice over-expressing SIRT1 relative to wild type controls of the same age. Protein expression was measured relative to the GAPDH band value. Protein expression was measured relative to the GAPDH band value. The results were normalized to the values for controls (100%). Data are means ± SEM. *p < 0.05 relative to controls (Student's *t* test). CO, control; p47, p47^phox^; RV, resveratrol, TGR, transgenic mice over-expressing SIRT1.

As indicated above, AII/AT1/NADPH-oxidase activity in the nigral region is higher in aged rats than in young rats; see [33] for details. As expected, levels of SIRT1 were increased significantly by treatment with resveratrol (Figure 2B). Therefore, in a second series of experiments, we investigated the effects of resveratrol on RAS activity in aged rats. Treatment with resveratrol induced a significant decrease in the expression of AT1 receptors, with no changes in AT2 receptor expression (i.e. a reduction in the AT1 to AT2 ratio), as well as a significant decrease in markers of NADPH-oxidase activation (i.e. p47^phox^ subunit expression) (Figure 4A). In young transgenic mice over-expressing SIRT1, we observed a significant decrease in levels of the NADPH-oxidase subunit p47^phox^, which reflected decreased levels of NADPH-oxidase activation. However, we did not detect any significant decrease in levels of AT1 receptors and AT2 receptors in young transgenic mice relative to young wild type mice (Figure 4B). In aged transgenic mice over-expressing SIRT1, we observed significant decrease in p47^phox^ and AT1 expression relative to aged WT mice (Figure 4C).

**Figure 5:**
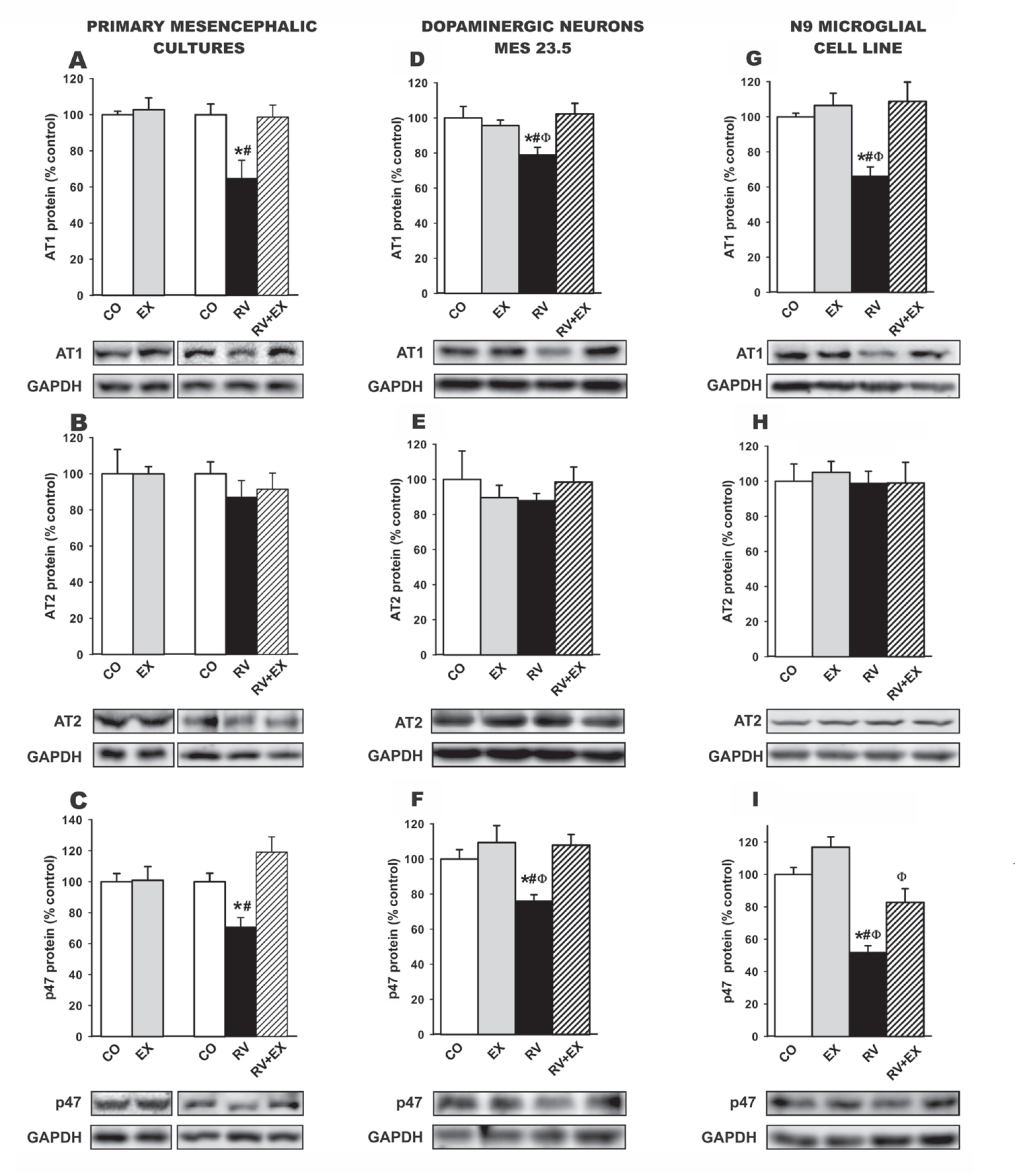
**Western blot analysis of changes induced by treatment with resveratrol (RV) and resveratrol plus the SIRT1 specific inhibitor EX527 (RV+EX)** in the expression of angiotensin receptors (AT1 and AT2) and NADPH-oxidase activation (i.e. p47^phox^ subunit expression) in primary (neuron-glia) mesencephalic cultures **A.**-**C.**, the dopaminergic cell line MES 23.5 **D.**-**F.** and the N9 microglial cell line **G.**-**I.**. The RV-induced decrease in AT1 and p47^phox^ subunit expression was inhibited by the SIRT1 specific inhibitor EX527. Protein expression was measured relative to the GAPDH band value. The results were normalized to the values for controls (100%). Data are means ± SEM. *p < 0.05 relative to controls, ^#^*p* < 0.05 relative to RV+EX, ^φ^*p* < 0.05 relative to EX (one-way ANOVA followed by Student-Newman Keuls). AII, angiotensin II; CO, control; EX, SIRT1 inhibitor EX527; p47, p47^phox^; RV, resveratrol.

We performed *in vitro* studies to identify the types of cells involved in the above-mentioned effects. WB studies in primary mesencephalic cultures (Figure 5A-5C), the dopaminergic neuron cell line MES 23.5 (Figure 5D-5F) and the N9 microglial cell line (Figure 5G-5I) revealed that treatment with resveratrol induced a significant decrease in AT1 receptor expression with no significant change in levels of AT2 receptors (i.e. a decrease in the AT1 to AT2 ratio), as well as decreased expression of p47 ^phox^ (i.e. NADPH-oxidase activation). All of these changes were inhibited by the simultaneous treatment with the SIRT1 specific inhibitor EX527, which indicates that SIRT1 activation plays a major role in the above described effects of resveratrol.

**Figure 6:**
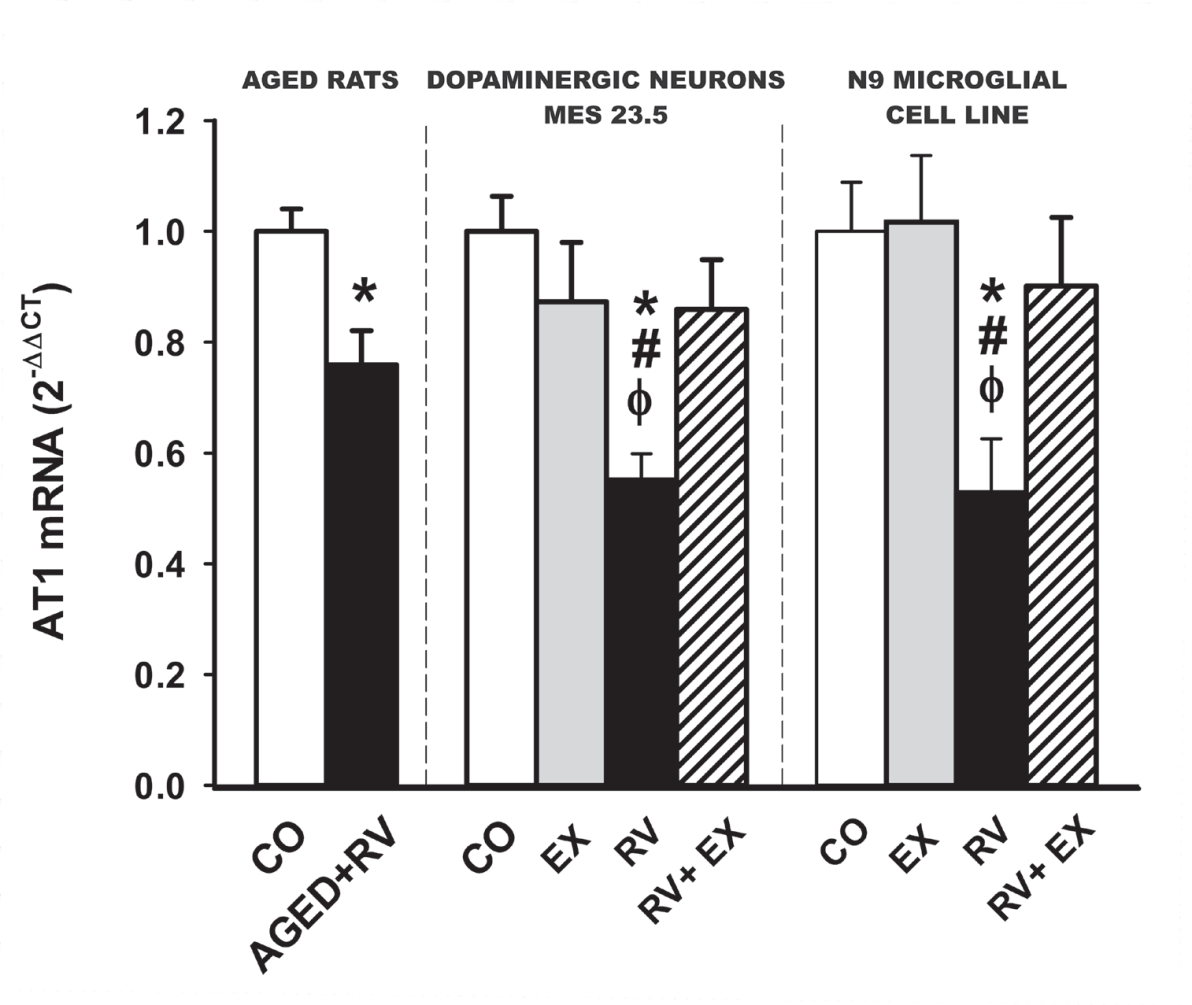
**Real-time quantitative RT-PCR analysis of changes in the expression of AT1** in the nigral region in aged rats treated with resveratrol or vehicle, and in cultures of the dopaminergic cell line MES 23.5 and the N9 microglial cell line treated with resveratrol or resveratrol plus the SIRT1 specific inhibitor EX527. The comparative cycle threshold values method (2 ^−ΔΔCt^) was used. The expression of each gene was measured relative to that of the housekeeping transcripts (β-Actin). Data are means ± SEM. *p < 0.05 relative to controls, ^#^*p* < 0.05 relative to RV+EX, ^φ^*p* < 0.05 relative to EX (Student's *t* test and one-way ANOVA followed by Student-Newman Keuls). CO, control; Ct, cycle threshold; EX, SIRT1 inhibitor EX527; RV, resveratrol.

As down regulation of AT1 receptors is a key finding of this study, the resveratrol-induced decrease in AT1 receptor expression was confirmed by quantitative RT-PCR in rats and cultures, which also revealed a resveratrol-induced decrease in AT1 mRNA expression. This indicates that the downregulation of AT1 receptor expression takes place at the transcriptional level and confirms the specificity of the results obtained with the AT1 antibody (Figure 6).

## DISCUSSION

In the present study, we observed the presence of SIRT1 in dopaminergic neurons and glial cells in the substantia nigra of rodents and in primary mesencephalic cultures and cell lines of dopaminergic and microglial cells. We also observed an important reciprocal regulation between SIRT1 and the local RAS both *in vivo* and *in vitro*.

### Effects of RAS on SIRT1 expression

Intraventricular administration of AII to young rats induced an increase in SIRT1 expression in the nigral region. The increase in SIRT1 expression may counteract AII-induced oxidative stress (OS). In several types of cells, SIRT1 stimulates resistance to oxidative stress through peroxisome proliferator activated receptor gamma coactivator 1α (PGC-1α) [38], FOXO [5, 39] and manganese superoxide dismutase (Mn-SOD) [6]; see for review [7]. In the present study, we have shown that SIRT1-induced downregulation of the AT1/NADPH-oxidase axis constitutes an important additional mechanism to counteract OS and inflammation (see below). In young rats, we also observed a lack of response of SIRT1 to chronic stimulation with AII (i.e. seven daily injections). This may be explained because a persistent increase in OS was found to mediate SIRT1 degradation and decreased SIRT1 expression [40, 41]; however, in this case, a decrease in SIRT1 expression below control levels is expected, and we observed no differences between young rats subjected to chronic stimulation with AII and untreated controls. A more interesting explanation is related to AII-induced desensitization of the AT1 receptor, which has been previously observed in different tissues and cell culture models [42-44]. Signal transduction by G-protein-coupled receptors is often accompanied by desensitization, which has been related to three major mechanisms [45]: 1) rapid uncoupling from G-protein, 2) sequestration of receptors into endosomal vesicles, and 3) downregulation of the total number of receptors. Further research is required to fully clarify the mechanism responsible for the lack of response of SIRT1 to chronic stimulation with AII. However, an interesting possibility may be related to a SIRT1-induced downregulation of AT1 receptors observed in the present study and discussed in the following section.

In aged rats, we observed a decrease in levels of SIRT1 relative to young control despite the fact that RAS activity and markers of OS and neuroinflammation are known to be enhanced [30, 33, 34]; a counterregulatory increase in SIRT1 levels may be expected, as observed in young rats. A decline in SIRT1-protein levels with aging has also been observed in other organs such as thymus and heart, although the responsible mechanisms remain unclear [46, 47]. It has been suggested that a decrease in nuclear SIRT1 activity may be the initial event, which may start the age-related mitochondrial decline and other age-related major degenerative changes [8], which may include the increased AT1 expression and NADPH-oxidase activity observed in aged animals (i.e. the loss of the counter-regulatory mechanism of SIRT1 on AT1/NADPH-oxidase described in the present study). In the substantia nigra of aged rats similar age-related inability to upregulate other compensatory mechanisms against OS and neuroinflammation has been observed [33, 34, 48]. In the present study, we also observed that the level of SIRT1-protein expression was higher in aged (18 month-old) wild type mice than AT1 KO mice of the same age, in which RAS activity and OS are known to be lower [23, 49, 50]. This suggests that in aged wild-type mice, higher RAS activity and OS lead to discrete compensatory upregulation of SIRT1 expression. A previous study showed that SIRT1 levels did not differ significantly in the kidneys of very old mice (aged 26-30 months) and AT1 KO mice of similar age [23], which suggests that very old WT mice have already lost the minimal capacity to upregulate SIRT1 expression in response to overactivation of the AII/AT1 pathway, and possibly also in response to other OS inducers; however, the differences in findings may also be related to the type of tissues used (i.e. kidney and brain). It is also interesting that no differences in SIRT1 expression was observed between young WT and young AT1 KO mice, which suggests that the AII/AT1 pathway does not upregulate SIRT1 expression under control physiological levels of AT1 activity (i.e. in young WT mice).

*In vitro* studies were performed to identify the cell types involved in the above-mentioned effects. SIRT1 expression was observed in neurons, astrocytes and microglia in cultures. However, the present studies of the substantia nigra revealed much lower SIRT1-protein expression in astrocytes than in neurons and microglia. Several recent studies have reported major effects of SIRT1 and SIRT1-activating compounds on the microglial neuroinflammatory response [16, 17, 51]. Neuroinflammation and microglial activation are known to play a major role in the progression of dopaminergic degeneration in PD models and PD [52-55]. Microglial cells also mediate major effects of RAS hyperactivity on dopaminergic neuron degeneration [26, 27, 56]. Therefore, we investigated the effects of AII treatment on the dopaminergic neuron cell line MES 23.5 and on the N9 microglial cell line. In both dopaminergic neurons and microglial cells, AII induced an increase in SIRT1 expression. Consistent with that observed after chronic stimulation with AII *in vivo*, the increase in SIRT1 expression was detected after 4 h but not after 16 h (see above). In previous studies, we observed that treatment with AII induced an increase in OS in dopaminergic neurons via activation of neuronal AT1 receptors and, particularly, via activation of AT1 microglial receptors, which exacerbated the microglial neuroinflammatory response, leading to the release of NADPH-oxidase-dependent superoxide and inflammatory cytokines, which act on dopaminergic neurons [26, 27, 56, 57]. The present *in vitro* experiments indicate that activation of the AII/AT1 leads to compensatory increases in SIRT1 expression in both dopaminergic neurons and microglial cells. This is consistent with several recent findings showing that SIRT1 and the SIRT1-activating compound resveratrol exert neuroprotection via inhibition of the microglial neuroinflammatory response [16-18, 51]. It is also consistent with the results of the present study, which show that resveratrol, via SIRT1, inhibits the pro-inflammatory and oxidative AII/AT1 pathway in neurons and microglial cells (see below).

### Effects of SIRT1 on RAS activity

The effects of SIRT1 on RAS activity were studied *in vivo* by treatment of rats with the SIRT1-activating compound resveratrol and in transgenic mice overexpressing SIRT1. Previous studies have shown RAS hyperactivity in the nigra of aged rats relative to young control rats; increased expression of AT1 receptors, decreased expression of AT2 receptors and increased NADPH-oxidase activity were observed in aged rats [33, 34, 48]. In the present study, treatment with resveratrol induced a significant decrease in RAS activity in aged rats (i.e. a decrease in AT1 receptor expression and NADPH-oxidase activity). In the nigral region of young transgenic mice, moderately increased SIRT1 expression and decreased expression of the NADPH-oxidase subunit p47 ^phox^, reflecting a decrease in NADPH-oxidase activity, were observed. A decrease in the expression of p47 ^phox^ in mice overexpressing SIRT1 is consistent with previous findings suggesting that inhibition of translocation of p47 ^phox^ is a major factor in the neuroprotective effects of SIRT1 and resveratrol, which did not exert neuroprotective effects in cultures from NADPH-oxidase-deficient mice [18, 58]. However, we did not observe any significant changes in the levels of angiotensin receptors relative to those in young control mice. This is consistent with previous findings showing that the SIRT1 transgenic mice were protected against high-fat-diet-induced increase in hepatic inflammatory cytokines but levels of cytokines were not different from WT mice under normal diet [59]. This is also consistent with the present findings in young AT1 KO mice, in which no significant changes in SIRT1 expression were observed. However, SIRT1 overexpression and treatment with the SIRT1-activating compound resveratrol counteract the abnormal increase in RAS activity observed in aged animals. This was confirmed in aged transgenic mice over-expressing SIRT1, which showed decreased AT1 and NADPH (p47 ^phox^) expression relative to WT mice of the same age.

The inhibitory effect of resveratrol and SIRT1 overexpression on the AII/AT1/NADPH-oxidase pathway was confirmed in primary mesencephalic cultures, in the dopaminergic neuron cell line MES 23.5 and in the N9 microglial cell line. Treatment with resveratrol induced a significant decrease in levels of AT1 and p47 ^phox^, and this effect was blocked by the simultaneous treatment with the SIRT1 inhibitor EX527, which is consistent with the major role of SIRT1 in the inhibitory effects of resveratrol on the microglial response [16-18, 51].

The exact mechanism responsible for the inhibitory effect of SIRT1 on the AII/AT1 pathway remains to be fully clarified. However, the present findings revealed a resveratrol-induced decrease in AT1 mRNA expression, which indicates that the downregulation of AT1 receptor expression takes place at the transcriptional level. SIRT1 stimulates resistance to oxidative stress through activation of several transcription factors including PGC-1α [38] and FOXO [5, 39]. However, it is also known that SIRT1 downregulates the activity of the transcription factor NF-κB [59, 60], which is particularly interesting for the present study. In microglial cells, we have recently shown a positive correlation between AT1 expression, NADPH activity, NF-κB and Rho kinase (ROCK), which may provide a basic means of coordinating the microglial neuroinflammatory response [56]: AII, via AT1 receptors, induces NADPH-oxidase activation and superoxide generation, which leads to NF-кB translocation and ROCK activation. ROCK activation in turn induces NADPH-oxidase activation via p38 MAPK through a feed-forward mechanism. Moreover, ROCK activation, via NF-кB, upregulated AT1 receptor expression in microglial cells through a feed-forward mechanism [56]. Several studies in different types of cells have shown that SIRT1 inhibits NADPH oxidase activation [58, 61]. Therefore, a SIRT1-induced decrease in NADPH activity may lead to inhibition of NF-κB and the positive feedback mechanism on AT1 receptors and other components of the microglial neuroinflammatory response [56]. Additional mechanisms may also be involved in SIRT1-induced inhibition of NF-κB and AT1 mRNA expression [7, 60].

In conclusion, the local RAS and SIRT1 both play major roles in the modulation of neuroinflammation, oxidative stress and aging-related cell degeneration, and particularly dopaminergic vulnerability to damage. The present results show that SIRT1 and the axis AII/AT1/NADPH-oxidase regulate each other in the modulatory process and that this mechanism is impaired in aged animals and it may be mitigated with Sirtuin-activating compounds such as resveratrol.

## MATERIALS AND METHODS

### Experimental design

Both *in vivo* and *in vitro* experiments were conducted. *In vivo* experiments were carried out with young adult (10-weeks old at the beginning of the experiments) and aged (18 to 20-months old) male Sprague Dawley rats, and male C57BL-6 wild type (WT), knockout (KO) and transgenic mice. A first group of mice were young adult (eight weeks old) and aged (18 to 20-months old) male WT C57BL-6 mice (Charles River, L'Arbresle, France), and young and aged homozygous C57BL-6 mice deficient for AT1a (the major mouse AT1 isoform and the closest murine homolog to the single human AT1; strain B6.129P2-Agtr1atm1Unc/J, Jackson Laboratory, Bar Harbor, ME, USA). A second group of C57BL-6 mice were young and aged transgenic mice, in which SIRT1 is moderately over-expressed under its own promoter, thereby following the physiological pattern of expression [59], and the corresponding WT controls. Rats and mice were housed under a 12 h light/dark cycle with ad libitum access to food and water. All experiments were carried out in accordance with Directive 2010/63/EU and Directive 86/609/CEE and were approved by the corresponding committee at the University of Santiago de Compostela. All rats and mice were anaesthetized with ketamine/medetomidine prior to undergoing surgery.

A first series of *in vivo and in vitro* experiments was undertaken to investigate the effects of increased RAS activity on SIRT1 levels. First, one group of young rats received one or seven intraventricular injections of AII (*n* = 24; see below) to investigate the effects of increased AII levels on SIRT1 levels in the nigral region. As RAS activity in the nigral region is known to be higher in aged rats than in young rats (i.e. increased activity in the AII/AT1/NADPH-oxidase pathway; see [33, 34] for details), we also investigated the levels of SIRT1 in aged rats (*n* = 6) relative to young rats (*n* = 6); in addition, aged rats were treated with the SIRT1-activating compound resveratrol to study the effects on levels of SIRT1 (*n* = 5) relative to untreated aged rats (*n* = 7). The effect of the AII/AT1 pathway on nigral SIRT1 levels was further investigated in young (*n* = 7) and aged (*n* = 6) mice deficient in AT1 receptors (AT1 KO mice), by comparison with young (*n* = 7) and aged (*n* = 6) WT mice. In previous studies, we observed that AII exerts pro-oxidative and pro-inflammatory effects by acting directly on dopaminergic neuron AT1 receptors and, particularly, on microglial cells; see [35, 36] for review. In the present study, we therefore investigated the *in vitro* effect of AII on SIRT1 levels in cultures of the dopaminergic neuron cell line MES 23.5 and the N9 microglial cell line.

A second series of *in vivo* and *in vitro* experiments was undertaken to investigate the effects of increased SIRT1 levels on the activity of the AII/AT1/NADPH-oxidase pathway. First, aged rats (i.e. rats with increased AII/AT1 activity) were treated with the SIRT1-activating compound resveratrol (*n* = 9). We then determined nigral levels of AT1 and AT2 receptor expression and p47^phox^ (a marker of activation of the NADPH-oxidase complex) relative to untreated aged rats (*n* = 11). We then investigated the expression of the same compounds in young (*n* = 7) and aged (*n* = 4) transgenic mice over-expressing SIRT1, relative to expression in the corresponding young (*n* = 7) and aged (*n* = 6) WT controls. We investigated *in vitro* the effects of treatment with resveratrol on primary mesencephalic neuron-glia cultures and on cultures of the dopaminergic neuron cell line MES 23.5 and the N9 microglial cell line. The role of SIRT1 activation in the resveratrol-induced effects was confirmed with the specific SIRT1 inhibitor EX527.

The brains were rapidly removed from rats and mice and the mesencephalon was sliced coronally (1 mm) with a tissue chopper. To isolate the nigral region (SNc), the individual 1mm tissue slides were dissected on a pre-cooled glass plate under a stereoscopic microscope. The substantia nigra compacta (SNc) was dissected according to [62], frozen on dry ice, and stored at −80°C until processed for western blot (WB) or real-time quantitative RT-PCR (RT-PCR) studies (see below). In addition, some rats (*n* = 4) were sacrificed, perfused with 4% paraformaldehyde (PFA) and processed for histology (see below). Cultures were also processed for WB and RT-PCR as detailed below. Brain sections of rat substantia nigra and different types of cultures were processed for immunofluorescence and confocal microscopy to confirm the presence of SIRT1 protein in the different types of cells investigated in the present study.

### Treatment of rats with intraventricularAII and resveratrol

Young adult rats were anaesthetized and divided into two groups. As it is known that AII does not cross the BBB under normal conditions, one group of rats was injected in the third ventricle (stereotaxic coordinates: 0.8 mm posterior to bregma, midline, 6.5 mm ventral to the dura, and tooth bar at 0) with a single injection of AII (5μg in 3 μl of sterile saline, *n* = 6; Sigma). The solution was injected using a 10 μl Hamilton syringe coupled to a motorized injector (Stoelting), at a rate of 0.5 μl/min. Another group of rats was injected with a 5 μg injection for seven consecutive days (*n* = 6). The consecutive injections were performed using a single cannula placed in the third ventricle during the whole injection period as described previously [63]. Control rats were injected with vehicle (*n* = 6 per group). Sixteen hours after the last AII injection the rats were killed and processed for western blotting (WB). The effective doses of AII and survival period were determined on the basis of our previous findings [63].

Resveratrol was freshly prepared in an emulsion of ethanol in olive oil (10 mg/ml). Rats were intraperitoneally injected twice a day every day for 10 days with either 15mg/Kg resveratrol (30 mg/Kg/day; R5010 Sigma Chemicals) or with vehicle [59-61]. Finally, rats were killed 1 h after the last injection. The dose of resveratrol was selected on the basis of earlier reports, which have demonstrated the neuroprotective properties of the compound at doses ranging from 20 to 50 mg/kg [64-66].

### Primary mesencephalic neuron-glia cultures

Ventral mesencephalic tissue was dissected from rat embryos of 14 days of gestation (E14). The tissue was incubated in 0.1% trypsin (Sigma), 0.05% DNase (Sigma, St. Louis, MO, USA) and DMEM (Invitrogen Life Technologies, Paisley, Scotland, UK) for 20 min at 37°C, and was then washed in DNase/DMEM and mechanically dissociated. The resulting cell suspension was centrifuged at 50 g for 5min, the supernatant was then removed carefully and the pellet was resuspended in 0.05% DNase/DMEM to the final volume required. The number of viable cells in the suspension was estimated by acridine orange/ethidium bromide staining, and cells were plated onto 35-mm culture dishes (Falcon, Becton Dickinson, Franklin Lakes, NJ, USA) previously coated with poly-L-lysine (100 μg/ml; Sigma) and laminin (4 μg/ml; Sigma). The cells were seeded at a density of 1.5 × 10^5^ cells/cm^2^ and maintained under control conditions [DMEM/HAMS F12/(1:1) containing 10% fetal bovine serum (FBS; BiochromKG, Berlin, Germany)]. The cell cultures were maintained in a humidified CO_2_ incubator (5% CO_2_; 37°C) for 7 days *in vitro* (DIV); the medium was totally removed on day 2 and replaced with fresh culture medium.

### N9 microglial and MES 23.5 dopaminergic neuron cell line cultures

The murine N9 microglial cell line was provided by Dr Paola Ricciardi-Castagnoli (Singapore Immunology Network, Agency for Science, Technology and Research, Singapore). The N9 microglial cells were cultured in Roswell Park Memorial Institute medium (RPMI 1640; Invitrogen, 21,875-091) supplemented with 10% FBS, 2mML-Glutamine (Sigma, G6392),100 U/ml penicillin, and 100 mg/ml streptomycin, and the cultures were maintained at 37°C, 95% air, and 5% CO_2_ in a humidified incubator [67]. The cells were then seeded onto 35-mm culture dishes (0.5 x10^6^ cells/ well) for analysis.

Dopaminergic MES 23.5 cells, a gift from Dr Wei-dong Le (Baylor College of Medicine, TX, USA), were cultured in DMEM/F12 containing Sato components growth medium supplemented with 2% fetal bovine serum (FBS), 100 units/ml penicillin, and 100 mg/ml streptomycin at 37°C in a humidified CO_2_ incubator (5% CO_2_, 95% air) [68]. For experiments, MES 23.5 cells were plated at a density of 0.5 × 10^5^/cm^2^ onto 35-mm plastic dishes, glass coverslips, previously coated with poly-L-ornithine (P-4638, Sigma; 10 mg/ml). Cells were stimulated to enhance differentiation by adding dibutyryl-cAMP (D0627, Sigma; 1 mM) to the supplemented growth medium, and they were grown to 80% confluence before the start of any treatment.

### Treatment of cultures

Cultures in the first series of experiments were treated with AII (100 nM) for 4 or 16 h. The most effective dose of AII was determined on the basis of our previous findings [26, 27]. Some cultures were treated with the AT1 receptor antagonist ZD 7155 (1 μM; Sigma) for 30 minutes before treatment with AII to confirm the involvement of AT1 receptors. Cultures in the second series of experiments were exposed to the SIRT1 activator resveratrol (50 μM; Cayman chemical) or the SIRT1specific inhibitor Ex 527 (Tocris; 1μM) [69, 70] for 30 min before treatment with resveratrol. The cells were then washed and processed for WB or RT-PCR.

### Western blotting of rat and mouse brains and cell cultures

Tissue from rat and mouse ventral midbrain and cultured cells (primary mesencephalic cultures, MES23.5 cells, N9 cells) were lysed in RIPA buffer containing protease inhibitor cocktail (P8340, Sigma) and PMSF (P7626, Sigma). Cell and tissue lysates were centrifuged, and the protein concentrations were determined by the BCA protein assay (Pierce 23225). Equal amounts of protein were separated by 10% Bis-Tris polyacrylamide gel, and transferred to nitrocellulose membranes. The membranes were incubated overnight with primary antibodies rabbit anti-SIRT1 (Millipore 07-131), goat anti-AT1 (sc-31181), rabbit anti-AT2 (sc-9040), and rabbit anti-p47^phox^ (sc-14015) from Santa Cruz Laboratories 1:200. The specificity of the antibodies was established in previous studies: AT1 sc-31181 [71], AT2 sc-9040 [72, 73], p47^phox^ sc-14015 [74], Sirt1 Millipore 07-131[75, 76]. In addition, the specificity of the antibodies was confirmed in our laboratory by preadsortion with the corresponding synthetic peptide antigen [77] and western blot analysis of lysates from HEK293 cells transfected with AT1 or AT2 tagged to fusion tail DDK (TA50011 from Origene; DDK tag: DYKDDDDK). The specifcity of the antibodies was confirmed by the presence of a predominant immunoreactive band in positively transfected lysates and the absence of this band in negative controls, which consisted of lysates transfected with empty vectors. The HRP conjugated secondary antibodies used were Protein A (NA9120V, GE Healthcare; 1:5000) and Chicken anti-mouse IgG (sc-2954, Santa Cruz Biotechnology Inc; 1:3000). Immunoreactivity was detected with an Immun-Star HRP Chemiluminescent Kit (170-5044, Bio-Rad) and visualized with a chemiluminescence detection system (Molecular Imager ChemiDoc XRS System, Bio-Rad). Blots were reprobed for anti-GAPDH (G9545, Sigma; 1:50000) as a loading control. In each sample, protein expression was measured by densitometry of the corresponding band and expressed relative to the GAPDH band value. The data were then normalized to the values of the control group of the same batch (i.e., they were expressed relative to the value obtained for the control; 100%) to counteract any inter-batch variability. Finally, the results were expressed as means ± SEM.

### RNA extraction and real-time quantitative polymerase chain reaction

Total RNA from the nigral region was extracted with Trizol (Invitrogen, Paisley, UK), according to the manufacturer's instructions. Total RNA (2.5 μg) was reverse-transcribed to complementary DNA with nucleoside triphosphate containing deoxyribose, random primers, and Moloney murine leukemia virus reverse transcriptase (200U; Invitrogen). Real-time PCR was used to examine the relative levels of type 1 angiotensin receptors. Experiments were performed with a real-time iCyclerTM PCR platform (BioRad, Hercules, CA, USA). β-Actin was used as housekeeping gene and was amplified in parallel with the genes of interest. The comparative cycle threshold values (Ct, cycle threshold) method (2 ^−ΔΔCt^) was used to examine the relative mRNA expression [78, 79]. A normalized value is obtained by subtracting Ct of β-actin from Ct of the interest (Δ Ct). As it is uncommon to use ΔCt as relative expression data due to this logarithmic characteristic, the 2 ^−ΔΔCt^ parameter was used to express the relative expression data [78, 79]. Finally, the results were expressed as mean values ± SEM. Primer sequences were as follows: for AT1a, forward 5′-TTCAACCTCTACGCCAGTGTG-3′, reverse 5′-GCCAAGCCAGCCATCAGC-3′, and for β-actin, forward 5′-TCGTGCGTGACATTAAAGAG-3′, reverse 5′-TGCCACAGGATTCCATACC-3′.

### Double immunofluorescence of rat substantia nigra and cell cultures

Rats used for double immunofluorescence were killed and perfused with 0.9% saline and then with cold 4% PFA in 0.1 M phosphate buffer (PB), pH 7.4. The brains were removed and subsequently washed and cryoprotected in the same buffer containing 30% sucrose, and finally cut into 30-μm sections on a freezing microtome. Cultures used for double immunofluorescence analysis were grown on glass coverslips and fixed with 4% paraformaldehyde (PFA) in Dulbecco's phosphate buffered saline (DPBS; pH 7.4) for 20 min. The cultures and brain sections were subsequently processed for double fluorescent labelling and incubated overnight at 4°C with the corresponding primary antibodies diluted in DPBS-1% bovine serum albumin (BSA) with 2% normal donkey serum (Sigma). The following primary antibodies were used: rabbit anti-Sirt1 (Millipore 07-131); mouse monoclonal anti tyrosine hydroxylase (TH; Sigma, 1:5000), as a marker of dopaminergic neurons; mouse anti CD11b (complement receptor-3, clone MRC OX42, Serotec; 1:50) as a marker of microglial cells; mouse anti NeuN (1:500, Millipore; as a neuronal marker), and mouse anti glial fibrillary acidic protein (GFAP, Millipore, 1:500), as a marker of astrocytes. The cultures were rinsed with DPBS and then incubated for 2h with the following fluorescent secondary antibodies: Alexa Fluor 568-conjugated donkey anti-rabbit IgG (1:200; Molecular Probes) and Alexa Fluor 488-conjugated donkey anti-mouse IgG (1:200; Molecular Probes). Finally, cultures were incubated for 30 min at RT with the DNA-binding dye Hoechst 33342 (3.9 × 10^−5^ M in DPBS) and coverslipped with Immumount (Thermo-Shandon). Colocalization of markers was confirmed by confocal laser microscopy (TCS-SP2; Leica, Heidelberg, Germany) and use of a sequential scanning method to prevent overlap. In all experiments, the control cultures, in which the primary antibody was omitted, were immunonegative for these markers. Tissue sections were counterstained with Hoechst 33342, mounted on gelatin-coated slides and coverslipped with Immumount. Colocalization of markers was confirmed by confocal laser microscopy.

### Statistical analysis

All data were obtained from at least three independent experiments and were expressed as mean values ± SEM. Two-group comparisons were carried out with the Student's t test and multiple comparisons with one-way ANOVA followed by Student-Newman Keuls. The normality of populations and homogeneity of variances were tested before each ANOVA. Differences were considered as statistically significant at *p* < 0.05. Statistical analyses were carried out with SigmaStat 3.0 from Jandel Scientific (San Rafael, CA, USA).
